# Uvularis muscle strengthening advancement in palatopharyngeal collapse in selected cases of snoring and obstructive sleep apnea: a randomized trial

**DOI:** 10.1007/s00405-025-09844-5

**Published:** 2025-12-22

**Authors:** Sherif M. Askar, Ali M. Awad, Ameer A. Abou-Sharkh

**Affiliations:** 1 Professor of Otorhinolaryngology, Head and Neck Surgery, Otorhinolaryngology, Head and Neck Surgery Department, Faculty of Medicine, Zagazig University, Zagazig, Egypt; 2 Assistant professor of Otorhinolaryngology, Head and Neck Surgery, Otorhinolaryngology, Head and Neck Surgery Department, Faculty of Medicine, Zagazig University, Zagazig, Egypt; 3 Lecturer of Otorhinolaryngology, Head and Neck Surgery, Otorhinolaryngology, Head and Neck Surgery Department, Faculty of Medicine, Zagazig University, Zagazig, Egypt; 4 Othman Bin Affan st, Zagazig city, Sharkia Governorate Egypt

**Keywords:** Obstructive sleep apnea, Uvularis muscle, Snoring, Palatal surgery, Uvulopalatopharyngoplasty

## Abstract

**Background and purpose:**

The uvularis muscles shorten, elevate, and retract the uvula and assist the levator veli palatini muscle in velopharyngeal closure. This work was designed to present the uvularis muscle advancement technique in a selected group of obstructive sleep apnea (OSA) patients, assess the surgical applicability, and report the surgical outcomes.

**Methods:**

Thirty-four adult patients with single-level OSA who showed a predominant anteroposterior pattern of collapse at the retropalatal region were stratified into two groups: Group A underwent modified anterior palatoplasty (MAP) only, and Group B had modified anterior palatoplasty with uvularis muscle advancement (MAP-UMA).

**Results:**

At 6–8 months, a highly significant improvement was reported as regards the mean apnea hypopnea index (MAP group; from 12.9 to 5.7; MAP-UMA group: from 19.1 to 4.0) and the mean lowest oxygen saturation (MAP group; from 89.9 to 93.7; MAP-UMA group: from 88.7 to 94.3; *P* < 0.000) in both groups; but the comparison of both groups showed non-significant differences (*p* > 0.05). The visual analog scale of snoring and Epworth Sleepiness Scale showed a significant (*p* < 0.05) reduction in both groups; the comparison between groups was highly significant towards MAP-UMA. According to Sher’s criteria, successful outcomes were reported in 27 patients (79.41%); MAP = 13 (76.47%), MAP-UMA = 14 (82.35%). The overall percentage of improvement (of the study group) was 78.65 ± 5.41%.

**Conclusion:**

MAP-UMA is an effective procedure in patients with OSA who show a predominant retropalatal collapse. UMA results in a significant improvement in snoring. It could be incorporated into multi-level surgery in OSA.

## Introduction

The uvularis muscle (UM) is located between the two laminae of the palatine aponeurosis. The muscle originates from the posterior nasal spine (of the palatine bone) and the upper aspect of the palatine aponeurosis. It passes posteriorly/superiorly to the swing (formed by the levator veli palatini muscle; LPM) and then inserts into the mucous membrane of the uvula. UM gains blood supply from the ascending palatine and the descending palatine arteries. A single UM contraction pulls the uvula to the same side; the contraction of the two muscles shortens, elevates, and retracts the uvula. By retracting the uvula (and thickening the middle part of the soft palate), the muscle assists LPM in elevating the levator aponeurosis to aid in velopharyngeal closure [1–5]. By reviewing the available English-language research, UM did not have the care it deserved among sleep surgeons who tend to handle (with other palatal muscles) as a single unit.

The most common site of obstruction in obstructive sleep apnea (OSA) patients is the retro-palatal region (RP) [5–11]. Although UM is small, it controls a critical area of the palate with uvular contraction; hence, it could have an important role in airway patency and uvular vibrations (a main factor in snoring). In this work, the authors hypothesize that the individual care of UM during palatal surgery for OSA could enhance surgical outcomes via strengthening the uvula, widening the airway, and reducing snoring.

Thus, this work presents the uvularis muscle advancement technique combined with modified anterior palatoplasty in a selected group of OSA patients with predominantly palatal collapse, assessing the surgical applicability and reporting the surgical outcomes.

## Patients and methods

### Settings

This prospective randomized interventional clinical study was conducted at the ENT surgery department from May 2020 to June 2025.

### Ethical consideration

The Zagazig University Review Board approved the methodology (Zag-IRB#1809). The study was conducted according to the Declaration of Helsinki on Biomedical Research Involving Human Subjects. Informed written consent was obtained from all patients.

### Inclusion and exclusion criteria

The study included adult patients (referred to ENT outpatient clinic) with OSA who had an apnea-hypopnea index (AHI) > 5, a body mass index (BMI) of ˂35 kg/m^**2**^, and were CPAP non-compliant. Assuming the probability of OSA in group A (modified anterior palatoplasty: MAP) is 46.2%, in group B (modified anterior palatoplasty with uvularis muscle advancement: MAP-UMA) 29%, confidence level 95%, and power 80% (estimated β = 20), the total sample size was 34 patients (17 in each group). During preoperative endoscopic preparation, the study group had a single-level palatal collapse (anteroposterior pattern).

Exclusion criteria included patients with other sites (multi-level) upper airway collapse (UAC) and macroglossia. Patients with a history of surgical intervention for snoring/OSA or neck pathologies were excluded.

### Preoperative preparation

The study group had attended polysomnography (PSG; in-lab level II SOMNO screen TM plus/SOMNO medics; Randersacker-Germany). PSG data included: AHI, the oxygen desaturation index (ODI: the average number of O_**2**_ desaturation episodes/hour of sleep), the lowest O_**2**_ saturation (LO_**2**_), and the percentage of sleep time with O_**2**_ saturation below 90% (T90%). On PSG, AHI was recorded in different sleep positions; patients were nominated either positional OSA (PP) or non-positional OSA (NPP) [3, 12, 13].

The study protocol began with a detailed sleep history and otorhinolaryngology examination, and then the oral examination followed (a clinical assessment of dentition, soft palate, and tonsil size grading 1–4). Friedman’s staging system (FAS) was employed [13]. All patients filled out the Epworth Sleepiness Scale (ESS). The 0–10 visual analog scale of snoring (VAS-s: 0 = no snoring, 10 = maximum snoring loudness) was applied for snoring assessment and was completed by the bed partners.

The positional awake (the patient is examined by naso-endoscopy (NE) and Müller’s maneuver (MM) in the sitting (MM-S) and supine position; MM-P) was performed, and details of UAC (site, degree, and pattern) were reported [13, 14].

Patients in this study were randomly (closed envelopes; also known as sequentially numbered, opaque, sealed envelope method, where treatment allocation is predetermined and placed in serially numbered, opaque, sealed envelopes) distributed in 2 groups: Group A (17 patients) underwent modified anterior palatoplasty (MAP) only and Group B (17 patients) had modified anterior palatoplasty with uvularis muscle advancement (MAP-UMA). Routine preoperative investigations followed.

DISE was performed in the theatre just before the induction of general anesthesia. The NOHL grading system was reported for DISE and NE. The scale reports the primary sites of UAC [15].

### Surgical techniques

**Fig. 1:**
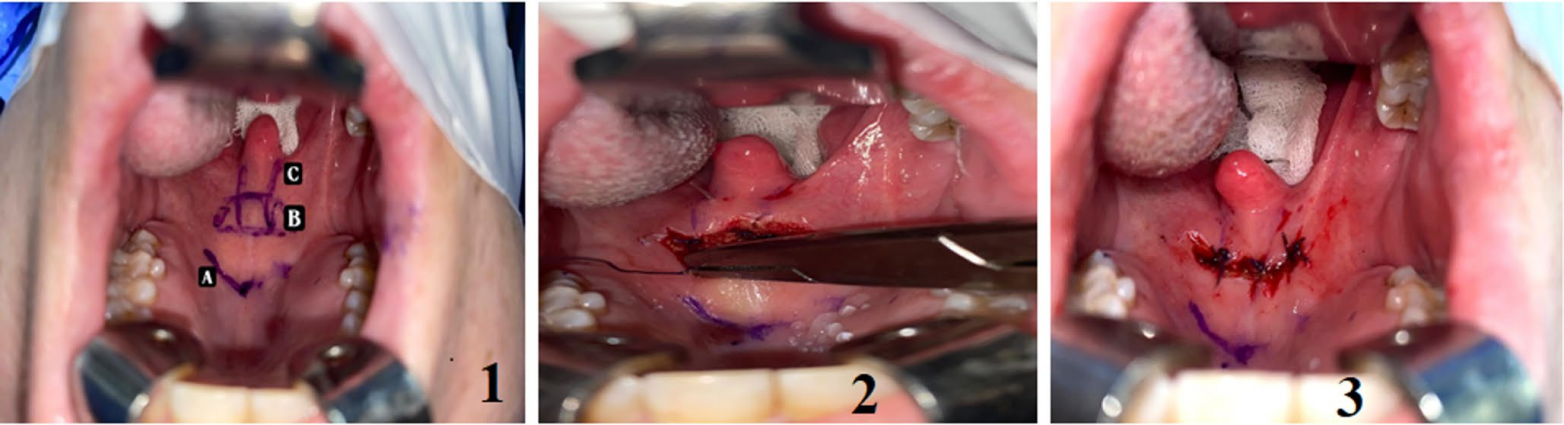
Design of modified anterior palatoplasty with uvularis muscle advancement:** A** (Junction of the soft and hard palate), **B** (Modified anterior palatoplasty), **C** (The line of the right uvularis muscle advancement stitch) Left uvularis muscle advancement stitch; note the tip of the needle Final view after modified anterior palatoplasty with uvularis muscle advancement (note the forward position of the uvula)

**Fig. 2:**
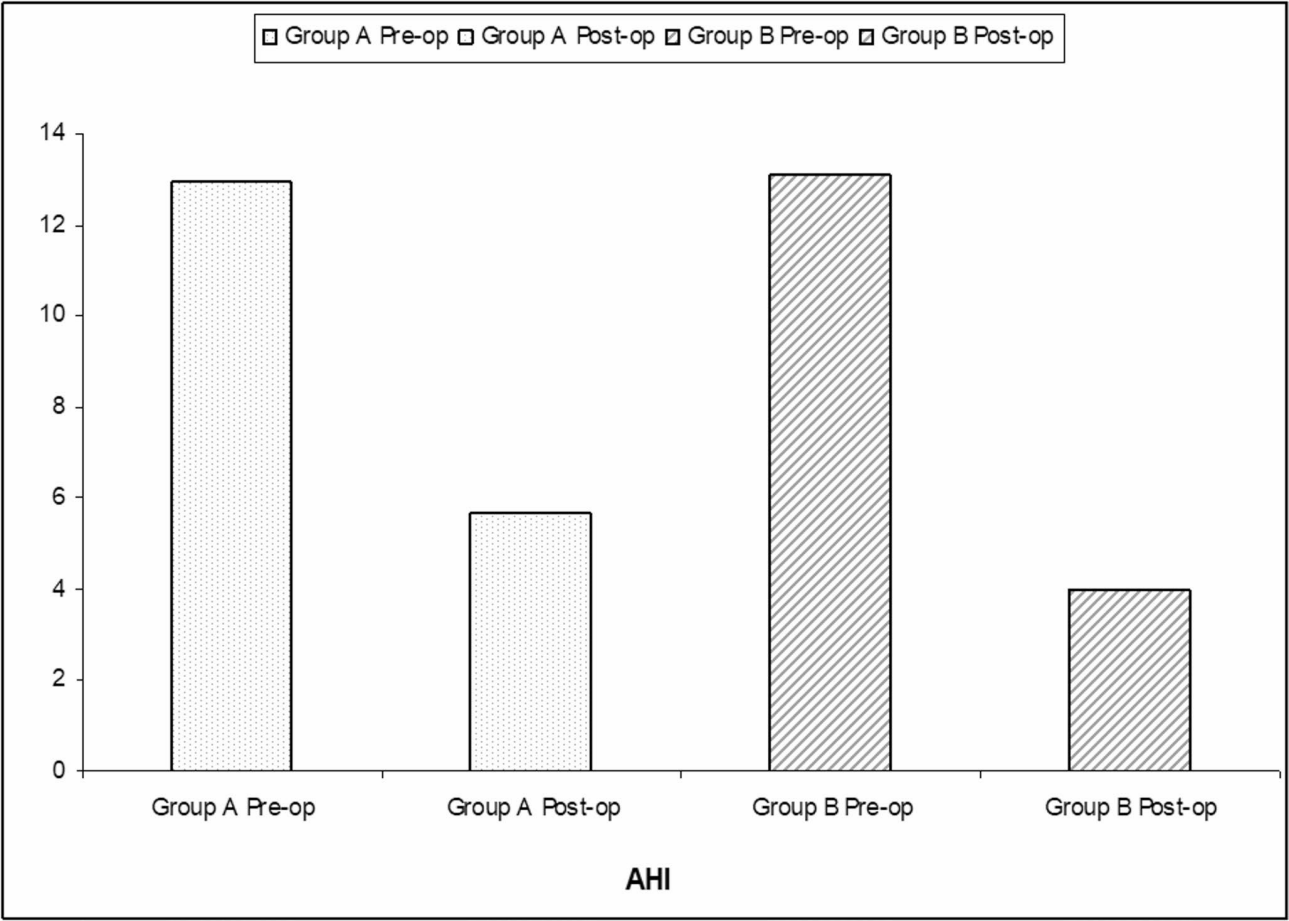
Mean AHI values for Group **A** (Modified anterior palatoplasty; MAP) and Group **B** (Modified anterior palatoplasty with uvularis muscle advancement; MAP-UMA); pre/post-operatively

All surgical techniques were performed under general anesthesia (with the patient in the tonsillectomy position). After DISE, the modified anterior palatoplasty (MAP) was performed as described [11]. MAP entailed the removal of a horizontal trapezoid strip of mucosa/submucosa of the soft palate 1 cm posterior to the hard/soft palate junction (5–7 mm in width). The anterior line of the trapezoid (40–45 mm) was longer than the posterior one (30–35 mm). The mucosa and sub-mucosal tissues were stripped to the muscle layer (using electro-cautery) with care not to injure the palatal muscles. The striped area was sutured using a round needle loaded with Vicryl 2/0. Initially, a midline simple suture was performed, followed by two horizontal mattress sutures in an anterolateral direction (forming an anterolateral parallelogram). The three sutures are passed through the soft palate, including the mucosa, submucosa, and muscles (in a multi-layer fashion) at multiple stations. Then, the three sutures were tied gently without over traction. In group B (MAP-UMA), uvularis muscle advancement (UMA) followed MAP (Fig. 1; 1); it was performed using a round needle loaded with Vicryl 2/0; the loaded needle was pushed (in the submucosa plane) from the palatine aponeurosis (starting from the anterior edge of the MAP), in the paramedian plane towards the ipsilateral side of the base of the uvula (Figs. 1 and 2). The needle is turned back, passed through the posterior edge of the MAP towards the starting point of the palatine aponeurosis, and then tied. An erected uvula is seen. After that, the MAP sutures are tied as described (Fig. 1).

The operative time was counted as the time for completion of MAP (A) and MAP-UMA (B). Patients were discharged 4–6 h after surgery and returned for examination after 7–10 days.

In the 6th −8th month after surgery, all patients underwent the postsurgical protocol: NE (with the Muller’s maneuver), and subjective outcome parameters were the snoring scores (SS), Epworth Sleepiness Scale (ESS) values, and postoperative pain (visual analog scale— VAS) values. Objective changes were presented via PSG data.

The surgical outcome followed Sher’s criteria for surgical success (≥ 50% decrease from the baseline AHI) [16]. Patient/partner satisfaction was also evaluated by a 0–10 visual analogue scale (VAS-sat: 0 = no patient satisfaction and 10 = maximum satisfaction). Bed partners completed VAS-s by 6 months postoperatively.

### Statistical analysis

Preoperative and postoperative statistics were reported using the SPSS program version 21.0 (SPSS Inc., Chicago, and Illinois-USA). Qualitative data were presented as frequencies and relative percentages. Quantitative data were represented as mean ± Standard deviation (SD). The comparison between pre- and post-operative quantitative variables was reported using non-parametric tests (Mann-Whitney, and Wilcoxon signed-rank tests). The power analysis estimation, including Beta value for the sample size, was 80%, with the confidence interval (CI) set at 95%. *P* < 0.001 was considered a highly significant result, *P* < 0.05 was significant, while P˃0.05 indicated non-significant.

## Results

### Patient characteristics

In this study, 121 patients were surgically eligible; 21 patients refused to participate in the study, 52 patients had exclusion criteria, and 14 patients did not complete the scheduled follow-up visits. The study group included 34 OSA patients (18 males and 16 females). Table 1 summarizes the basic data of the study group. The tonsil size was reported as grade I in all of them (100%). On FAS, 29 patients were stage I (85.29%), and 5 (14.71%) were stage II. PSG showed that all participants were PP (100%). During preoperative MM-S, MM-P, and DISE, all patients showed single-level palatal collapse (anteroposterior pattern). All patients reported early grades of collapse (NOHL scale grade 1–2; mean grade of 1.33 ± 0.42 for MM-S, 1.46 ± 0.05 for MM-P, and 1.51 ± 0.18 for DISE). All patients were recorded as PP. The follow-up period ranged from 13 to 22 months (mean = 16.33 ± 2.71). The mean operative time was 23.72 ± 0.08 min (range 19–29).

**Table 1:** Basic data of the study group (*N* = 34 patients)

Variables	Range	Mean ± SD/%
Age (years) MAP-UM MAP	21–38 years 21–36 23–38	30.2 ± 5.4 29.4 ± 3.05 32.1 ± 4.3
AHI MAP-UM MAP	9.64–16.42 10.2–16.42.2.42 9.64–16.64	12.94 ± 3.57 13.09 12.92
BMI (kg/m^**2**^) MAP-UM MAP	26.8–30.6 27.1–30.6 26.8–29.7	28.64 ± 2.32 29.05 ± 4.02 28.05 ± 3.54
NC (centimeters) MAP-UM MAP	39–44 39–44 39–43	41.35 ± 1.05 41.06 ± 2.13 40.84 ± 0.63

SD = Standard deviation; N: Number of participants; AHI: Apnea Hypopnea Index; BMI: Body Mass Index; NC: neck circumference (cm)

### Outcome

The mean operative time was 20 ± 2.52 (A: MAP), and 24 ± 1.78 (B: MAP-UMA); the difference is considered statistically significant (95% confidence interval of this difference: From − 3.5242 to −0.4758; *P* = 0.0117; t = 2.6728).

In both groups, postoperative pain was mild and was significantly improved by the 7th day; patients regained a normal diet within 2 weeks (range = 6–15 days; mean = 9.33 ± 1.03). No early (infection, bleeding, wound dehiscence) or late complications (voice changes, velopharyngeal incompetence, globus, taste disturbance) were reported. At 9–12 months, no swallowing/phonatory problems were reported. Regarding BMI and neck circumference, no significant changes were recorded (P˃0.05). None of the participants reported worsening AHI.

In both groups, postoperative PSG was requested after 6–8 months. All patients remained PP. Pre vs. postoperative PSG data showed highly significant improvement in the mean AHI, LO_**2**_, ESS, and VAS-snoring (P˂0.0001). Regarding hypoxia parameters, ODI and T90 showed highly significant improvements (Table 2; Figs. 2).

**Table 2:** Comparison of studied groups (pre/post-operative)

Item	Group A Pre-op Mean	Group A Post-op Mean	Group B Pre-op Mean	Group B Post-op Mean	IAG-*P*)	SG (Intra-Group)	IEG-*P*	SG (Inter-Group)
**AHI**	12.92	5.64	13.09	4.01	< 0.0001	HS	0.085	*P* = 0.118: NS
**LO**_**2**_	89.87	93.67	88.68	94.31	< 0.0001	HS	0.110	*P* = 0.261: NS
**T90%**	45.41	32.74	41.65	26.54	< 0.0001	HS	0.015	*P* = 0.024 Significant
**ODI**	16.03	6.03	16.71	4.39	< 0.0001	HS	0.072	*P* = 0.172: NS
**Snoring** **Index**	22.44	9.00	23.32	3.56	< 0.0001	HS	< 0.0001	*P* < 0.001: HS
**VAS-s**	6.88	2.87	7.64	0.69	< 0.0001	HS	< 0.0001	*P* < 0.001: HS

Data were expressed as a range, and mean; VAS-s: the visual analog scale of snoring; ODI: Oxygen desaturation index; T90: time below 90%; AHI: apnea-hypopnea index, LO_2_: Lowest oxygen saturation; *p* > 0.05 was considered no significant; SG: Level of significance; HS: highly significant; NS: non-significant; IAG-P: intragroup p-value (pre vs. post); IEG-P: inter-group p-value (postop Group A: modified anterior palatoplasty vs. B: modified anterior palatoplasty with uvularis muscle advancement)

**Fig. 3:**
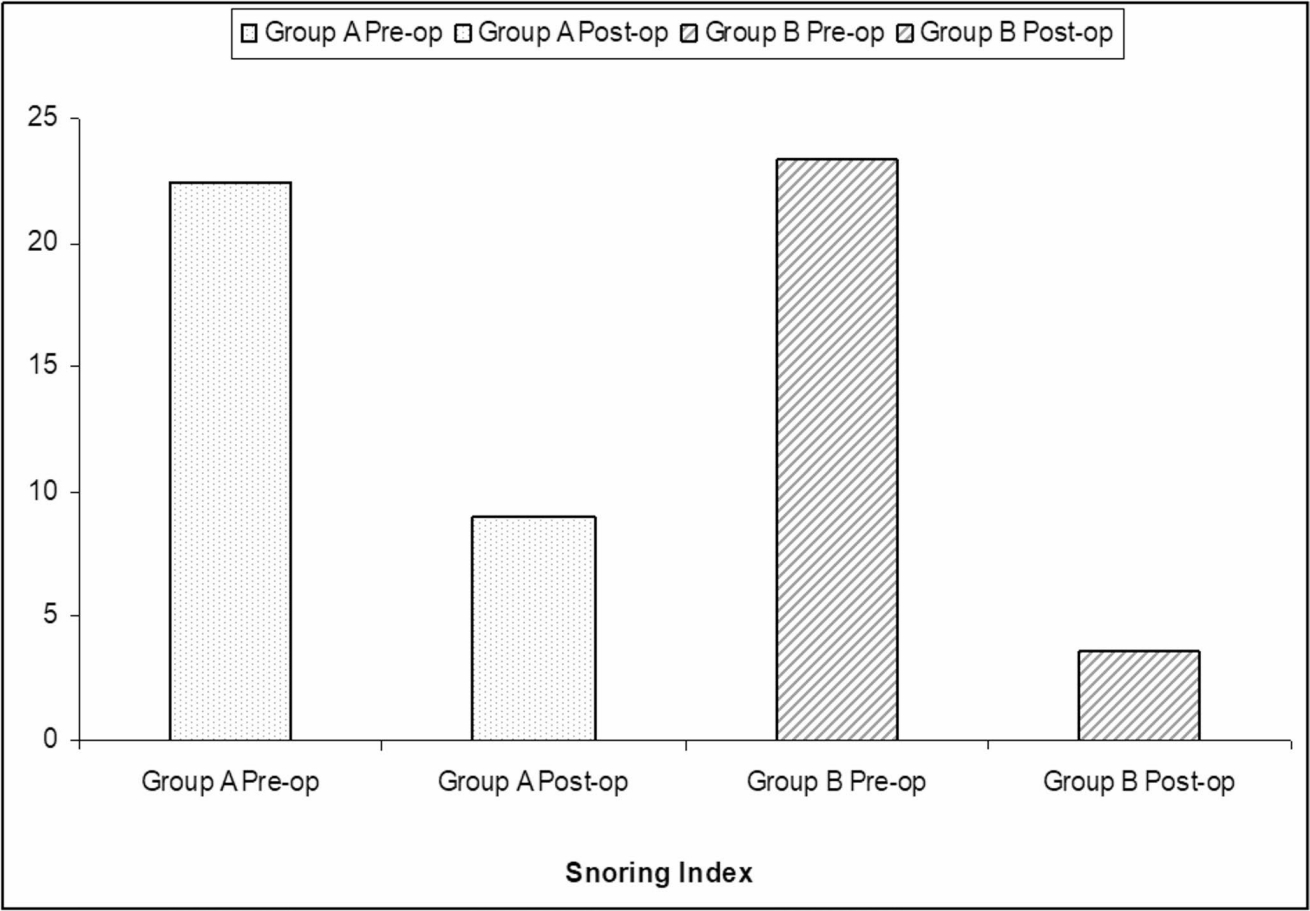
Mean snoring index values for Group (Modified anterior palatoplasty; MAP) and Group **B** (Modified anterior palatoplasty with uvularis muscle advancement; MAP-UMA); pre/post-operatively

The comparison of both groups showed non-significant differences regarding AHI, LO_**2**_, and ODI. Noticeably, T90% reported a significant difference (tendency towards MAP-UMA group). VAS-s and ESS showed highly significant differences (P˂0.0001, t = 8.6391) with a tendency towards MAP-UMA. VAS-sat showed highly significant improvements in both groups (*P* < 0.0001); on comparison of both groups, VAS-sat reported highly significant differences (P˂0.0001, t = 9.2401) with a tendency towards MAP-UMA (Table 2; Fig. 3).

According to Sher’s criteria, successful outcomes were reported in 27 patients (79.41%); MAP = 13 (76.47%), MAP-UMA = 14 (82.35%). The overall percentage of improvement (of the study group) was 78.65 ± 5.41%.

## Discussion

Modern concept of sleep surgery depends on providing rigid support to the collapsible upper airway soft tissues via the implementation of anatomically based, functional, muscle relocation and less-destructive procedures. A successful surgical plan depends on accurately determining the sites of collapse and identifying the anatomical locations amenable to surgical treatment. For that task, different tools exist, but endoscopic examination (awake or DISE) is considered the best, dynamic analysis of airway collapsibility [9–11, 16].

The retro-palatal region (RP) is the most common site of obstruction in OSA patients; moreover, palatal targeting surgeries are the most widely established procedures for snoring and OSA [8–12, 18]. The ideal surgical procedure for the palatal collapse should be easily performed, effective (good outcomes and consistent results among different surgeons), anatomically based, less ablative, and associated with minimal morbidities [4–11]. It should be performed within a reasonable short time (as it could be employed in multi-level surgery). Such an ideal surgery should be performed with minimal muscular trauma. Moreover, the budget should be considered. Unfortunately, such a perfect technique is yet to be found.

The interest in surgery on the palate in cases of OSA began in 1963 by the description of UPPP by Ikematsu; later, Fujita popularized the procedure. Unfortunately, significant comorbidities and limited success rates limited the application of UPPP with subsequent designs of less invasive and more functional techniques. Different palatal stiffening techniques were advocated, including injection (cautery-assisted) stiffening, snoreplasty, velo-uvulo-pharyngeal procedure, and radiofrequency ablation. These techniques aim to induce fibrosis and stiff scar tissue by removing redundant soft tissue. In the beginning, good results were reported, but with wide application, the effectiveness of the procedure and the patient’s satisfaction were debatable [17–22].

In 2009, anterior palatoplasty was described; the procedure entailed anterosuperior advancement of the soft palate. The procedure has high surgical success rates (86%). The double suspension suture technique (DSS) was designed to widen the oropharynx at two levels. DSS is rapid, simple, reversible, less costly, and has reported good surgical outcomes with minimal comorbidities [19]. The modified anterior palatoplasty (MAP) was presented in 2018. MAP entailed the removal of a trapezoid strip of soft palate mucosa (and sub-mucosa); the mucosa was sutured in an anterolateral parallelogram direction using Vicryl 2/0 multi-layer multi-station mattress sutures. Unlike the Pang procedure, MAP did not employ lateral cuts in the soft palate, and the uvula was not jeopardized. In that way, MAP could significantly open the retropalatal area via transposing the soft palate in an anterolateral direction, thus enlarging the anteroposterior axis of the retropalatal region [11].

Despite the availability of many techniques that could improve OSA, the problem of loud snoring might exist. Unfortunately, snoring is considered the main symptom of OSA, especially among young generations, and could harm the social life of the family. Thus, authors tend to combine multiple steps to control that issue [1, 8–11, 22, 23].

The current surgical technique (MAP-UMA) was inspired by previous work; it could be considered as an extension of MAP. The technique follows the concept of palatal surgery: the controlled induced fibrosis splints the palate (thus preventing collapse at the retropalatal airway space) in a less invasive manner. The technique is conservative, has minimal comorbidities, and was performed within a relatively short time.

MAP-UMA could be considered physiological as it deals with a functional problem (the increased collapsibility of the RP). The physiology-based correction of tissue collapsibility can be more effective by considering the anatomical characteristics of the main muscular components of the palate (non-destructive) and the transposition of these muscles in two different planes: antero-superiorly and antero-posteriorly. Postoperative pain and swallowing complaints were minimal (with MAP and MAP/UMA) and tolerable; this can be attributed to the conservative nature of the technique (no muscle cuts) and the completely mucosa-covered wound.

Although UM is small, its function is important in breathing and phonation; it controls a critical area of the upper airway (and the uvula) with an evident role in snoring. In literature, authors did not pay the deserved attention to the muscle. Uvularis advancement is suggested to enhance fibrous tissue formation (in the line of UM) thus, strengthening the muscle action with the resultant more widening of the airway, less uvular vibration, and hence less snoring.

### The improvement in PSG

The current study reported highly significant improvements in all PSG parameters (in both groups), including AHI and LO_**2.**_ It also showed highly significant improvements in all hypoxia-related parameters (ODI and T90%). This result indicates that both techniques were highly effective for patients with mild (and early moderate) OSA. However, the comparison of postoperative PSG findings between both groups was non-significant, suggesting comparable overall effectiveness in resolving OSA and improving general oxygenation. Noticeably, the results reported a statistically significant improvement in postoperative T90% in Group B (MAP-UMA) compared to Group A (MAP) indicating better oxygenation during sleep in MAP-UMA.

Tschopp et al. compared the effectiveness of different techniques of palatopharyngeal surgery in patients with OSA. They reported that, AHI responder rate was about 51% being favourable for conservative cold steel, muscle relocation sutures. Moreover, they documented unchanged surgical effectiveness for 3 years [19, 21]. Our results might match their results especially that the current technique is less traumatic, and tissue re-directing.

### Snoring improvement

The interesting findings of the current study are that the postoperative improvements regarding ESS and VAS-s were highly significant (with a noticeable tendency towards group B; MAP-UMA). This data could reflect the positive influence of UMA that would potentially enhance the final surgical outcome and overall patient satisfaction. Because of the importance of snoring in OSA patients, VAS-s was suggested as a surgical success parameter for OSA interventions (the sleep goal) [20]. Noticeably, patient/partner satisfaction was highly significant in both groups, with a tendency towards MAP-UMA.

Snoring is often described as the hallmark symptom of OSA and is the annoyance that may justify ridicule of the offending partner. Snoring has deleterious effects on human relationships and family stability. Surgical consultation is usually prompted by the bed partner; hence, solutions are primarily directed at the partner’s perspective and the social relationships. In the current work, ESS and VAS-s were significantly improved after MAP-UMA; moreover, there was a highly significant improvement on comparison to MAP alone. This might reflect the efficacy of UMA on snoring.

Finally, MAP-UMA is a cost-effective procedure; it requires no special equipment, uses less expensive materials/sutures, and a relatively short hospital stay. The technique has a short operative time (5 min); the reported improvement regarding snoring are worthy despite the time. It could be easily incorporated into multi-level surgery in advanced cases of OSA.

The current study shows limitations: (1) The relatively small sample size may limit the power of the biostatistics and the detection of subtle effects. (2) The short follow-up period. Also, it reports on a single team/institution experience. Longer follow-up studies are recommended for the proper evaluation of the technique and long-term outcomes.

## Conclusion

MAP-UMA is an effective procedure in patients with OSA who show a predominant retropalatal collapse. UMA results in a significant improvement in snoring. It could be easily incorporated into multi-level surgery in advanced cases of OSA.
